# Overexpression of SSR2 promotes proliferation of liver cancer cells and predicts prognosis of patients with hepatocellular carcinoma

**DOI:** 10.1111/jcmm.17314

**Published:** 2022-04-28

**Authors:** Fengsui Chen, Chen, Jielong Wang, Shi‘an Zhang, Mengxue Chen, Xia Zhang, Zhixian Wu

**Affiliations:** ^1^ Department of Hepatobiliary Disease 900 Hospital of the Joint Logistics Support Force Fujian Medical University Fuzhou Fujian P.R. China; ^2^ Department of Hepatobiliary Disease 900 Hospital of the Joint Logistics Support Force (Dongfang Hospital) Xiamen University Fuzhou Fujian P.R. China; ^3^ Fuzhou Hospital of Traditional Chinese Medicine Fuzhou Fujian P.R. China

**Keywords:** bioinformatics, hepatocellular carcinoma, prognosis, proliferation, SSR2

## Abstract

Signal Sequence Receptor Subunit 2 (SSR2) is a key endoplasmic reticulum gene involved in protein folding and processing. Previous studies found that it was upregulated in several cancers, but its precise role in hepatocellular carcinoma (HCC) remains unclear. To have a better understanding of this gene in HCC, we examined the expression of SSR2 in HCC tissues by analysing The Cancer Genome Atlas (TCGA) data and immunohistochemistry. We also assessed the association between SSR2 expression and clinicopathological characteristics of HCC patients and patient survival. Potential function of SSR2 was predicted through GSEA and protein–protein interaction analysis. MTT, flowcytometry, transwell and a nude mice xenograft model were employed to investigate the biological functions in vivo and in vitro. The results showed that the expression of SSR2 was significantly increased in HCC tissues, and SSR2 expression was associated with several clinical characteristics. In addition, patients with higher SSR2 expression had poorer survival. Enrichment analysis suggested that SSR2 was probably involved in biological process or signalling pathways related to G2/M checkpoint, passive transmembrane transporter activity, ATF2_S_UP. V1_UP and ncRNA metabolic process. Further experimental study showed that SSR2 knockdown inhibited cell proliferation, migration and invasion ability and promoted apoptosis and cell cycle arrest in vitro. Moreover, downregulation of SSR2 also repressed the growth of HepG2 cells in vivo. In conclusion, our study suggests that SSR2 may act as an oncogene in HCC.

## 1 INTRODUCTION

Hepatocellular carcinoma (HCC) is the dominant primary liver cancer, and accounts for 90% of patients with liver cancer.^1^ There are approximately 850,000 new liver cancer cases and 800,000 deaths worldwide each year.^2^ For early HCC, surgical resection and liver transplantation are recommended treatments.^3^ However, due to the difficulty in early diagnosis, a majority of patients are ineligible to surgical resection upon diagnosis. Multikinase inhibitors sorafenib and lenvatinib are the standard therapies for patients with advanced HCC, but with limited clinical efficacy.^4^ Therefore, novel biomarkers are necessary for timey diagnosis and effective treatment in HCC patients.

The signal sequence receptors (SSRs) are glycosylated endoplasmic reticulum (ER) membrane receptors associated with protein translocation within the ER membrane.^5^ Signal sequence receptor subunit 2 (SSR2), as one of the important subunits of SSR, was abundant in mammals. Studies have shown that SSR2 regulated the neural differentiation and zebrafish embryogenesis.^6^, ^7^ In recent years, SSR2 has been implicated in the progression of gastric cancer^8^ and melanoma.^9^, ^10^ In 2020, Hong et al. reported that SSR2 promoted the HCC metastasis by modulating epithelial–mesenchymal transition (EMT).^11^ However, a comprehensive role of SSR2 in HCC remains unclear.

In this study, we aimed to reveal the significance of SSR2 in HCC by using a variety of bioinformatics tool, in vitro experiments and a nude mice xenograft model.

## 2 MATERIALS AND METHODS

### 2.1 Data source and preprocessing

Gene expression data of TCGA‐ALL (included 730 precancerous and 10363 tumour tissues) and TCGA‐LIHC (included 50 precancerous and 374 tumour tissues) were collected from TCGA projects (https://portal.gdc.cancer.gov/).^12^ RNA‐seq data in level 3 HTSeq‐FPKM format of TCGA‐LIHC project was transformed into TPM (transcripts per million reads) by log2 for further analyses. Duplicate samples were removed for data filtering. Unavailable or unknown clinical features were regarded as missing values. For statistical analysis and visualization, ggplot2 version 3.3.3 were widely used. The study fully complied with publication guidelines stated by TCGA (https://cancergenome.nih.gov/publications/publication.guidelines).

### 2.2 SSR2 differential expression and survival analysis in the TCGA database

Boxplots and scatter plots were employed to generate differential expressions of SSR2 in TCGA using disease state (tumour or normal, TNM, pathological stage, histological stage and vascular invasion) as the variables. Statistical ranking for SSR2 expression above or below the median value was defined into SSR2‐high or SSR2‐low, respectively. The relationship between clinicopathological parameters and SSR2 was analysed with the Wilcoxon signed‐rank sum test and logistic regression. Cox regression and the Kaplan–Meier method were used to analyse the association between clinicopathological characteristics and the overall survival (OS), progression‐free interval (PFI) and disease‐specific survival (DSS) of TCGA‐LIHC patients. Multivariate Cox analysis was used to compare the influence of SSR2 expression on survival along with other clinical characteristics. The detailed procedure was as follows: RNA‐seq data in level 3 HTSeq‐FPKM format from the TCGA (https://portal.gdc.cancer.gov/) LIHC (hepatocellular carcinoma) project was filtered by removing duplicate samples and converted into TPM (transcripts per million reads) format. R (version 3.6.3) packages survival (version 3.2‐10) and survminer (version 0.4.9) were then employed for statistical analysis of survival data and subsequential visualization, respectively.

### 2.3 Analysis of DEGs between SSR2‐high and SSR2‐low expression GC groups

DEGs between SSR2‐high and SSR2‐low patients from TCGA‐LIHC datasets were determined by the DESeq2 (3.8) package, and the unpaired Student's *t*‐test was employed for statistical analysis. Genes with the adjusted *p*‐value <0.05 and the absolute FC > 1.5 were considered to be statistically significant. The DEGs between SSR2‐high and SSR2‐low patients were presented in volcano plot and heat map.

### 2.4 GSEA enrichment analysis

An ordered list of SSR2‐related DEGs was firstly generated based on their association with SSR2 expression by GSEA method.^13^ The expression level of SSR2 was a phenotype label. The number of gene set permutations were 1500 times for each analysis. The statistical significance of pathways was set as a normal *p*‐value <0.05 and an FDR q‐value < 0.25. Statistical analysis and graphical plotting were conducted using R package cluster Profiler. The diagnostic performance of SSR2 was estimated using receiver operating characteristic (ROC) curves.

### 2.5 Protein–protein interaction (PPI) network construction

To investigate the protein interactions between SSR2 and other proteins, a SSR2‐related PPI network was established via the Search Tool for the Retrieval of Interacting Genes/Proteins (STRING) database (https://string‐db.org/) with PPI pairs interaction score > 0.9.^14^

### 2.6 Cell culture and transfection

HepG2 and Huh‐7 cell lines were purchased from Cell Bank of Shanghai Institutes for Biological Sciences (Chinese Academy of Sciences) and cultured in Dulbecco's minimal essential medium (Gibco, USA) supplemented with 10% FBS (Gibco, Rockville, US). Cells were maintained at an incubator with the humidified atmosphere (37°C, 5% CO_2_). As for transfection of small interference RNAs, both cells were firstly seeded in 6‐well plates and then transfected with lipofectamine 2000 (Invitrogen, USA) 24 h later according to the manufacturer's procedures. The detailed sequences were as follows: siSSR2‐1: 5′‐GGUUCCAUCGUGAAGCCAUTT‐3′ (sense), 5′‐AUGGCUUCACGAUGGAACCAA‐3′ (anti‐sense); siSSR2‐2: 5′‐CCCUCCUCUCCCAAGAAAUTT‐3′ (sense), 5′‐AUUUCUUGG GAGAGGAGGGCT‐3′ (anti‐sense); siSSR2‐3: 5′‐GGUACUCCAGCAAGAGGAATT‐3′ (sense), 5′‐UUCCUCUUGCUGGAGUACCAC‐3′ (anti‐sense); siCtrl: 5′‐ UUCUCCGAACGUGUCACGUTT‐3′ (sense); 5′‐ACGUGACACGUUCGGAGAATT‐3′ (anti‐sense). pLKO.1‐shSSR2‐2 and pLKO.1‐shCtrl vectors were then constructed for further animal experiments.

### 2.7 RNA isolation and qRT‐PCR

Total RNA was extracted by TRIzol^®^ Plus RNA Purification Kit (Invitrogen, USA) and reversely transcribed by SuperScript™ III First‐Strand Synthesis SuperMix (Invitrogen, Switzerland). The mRNA expression was then determined by quantitative real‐time PCR (qRT‐PCR) with Power SYBR^®^ Green PCR Master Mix (Applied Biosystems, USA) as previously described.^15^ GAPDH was used as internal reference controls. The specific primer sequences were as follows. SSR2: 5′‐CTTCACCTCGGCAACAATTACT‐3′ (forward); 5′‐ GGGGAGAATCGC CTGTCAAAC‐3′ (reverse); GAPDH: 5′‐CCATGACAACTTTGGTATCGTGGAA ‐3′ (forward); 5′‐GGCCATCACGCCACAGTTTC ‐3′ (reverse). All experiments were carried out according to the manufacturers’ protocols and data expressed as bar in the mean ± SD in triplicate.

### 2.8 Western blot analysis

Cultured cells were lysed using Total protein extraction kit (including protease inhibitor cocktail). BCA Protein Assay Kit (Beyotime, China) was employed to determine the protein concentrations in each sample. Proteins were then separated in 10% separation gel and 5% concentration gel and transferred to PVDF membranes (Merck Millipore, Burlington, USA). After blocking in TBST containing the skimmed milk, PVDF membranes were incubated with primary antibodies against SSR2 (10278‐1‐AP) and GAPDH (ab181602). Then, the membraned were incubated with goat anti‐mouse IgG‐HRP secondary antibody (31160). The dilution of primary antibodies was as follows: SSR2 (1: 500, Proteintech, Chicago, US); GAPDH (1: 10000, Abcam, Cambridge, UK); secondary antibody (1:5000, Thermo Pierce, Waltham, US). SuperSignal^®^ West Dura Extended Duration Substrate was finally carried out for signal detection.

### 2.9 Cell proliferation assay

For cellular proliferation analysis, Cell Counting Kit‐8 (CCK‐8) was employed as described previously.^16^ In brief, the transfected HepG2 cells were plated in 96‐well plates at 2 × 103 per well. After culture for 1, 2, 3, 4 and 5 days, CCK‐8 was added to each well, and the data were recorded at the optical density (450 nm) on Infinite F50 (Tecan, China). At least three individual experiments were performed.

### 2.10 Cell cycle and apoptosis assay

Cell cycle and apoptosis assay were performed on cells transfected with siSSR2 or siCtrl to determine whether SSR2 regulates the growth phase and apoptosis of liver cancer cells. Cells were trypsinized, centrifuged at 300 × g (1000 rpm) for 5 min and resuspended in complete medium to form a cell suspension (1 × 10^6^ cells/ml) and fixed with 70% ice‐cold ethanol for 30 min. Then, the cells centrifuged, washed and resuspended in 250 μl PBS contained 10 μl of DNase free RNase (final concentration is 1‰). After PBS washing and incubation with pyridine iodide (PI, 0.05 mg/ml) for 30 min, cells were incubated for an additional 15 min in the dark. The fluorescence of PI was then measured using FACS Calibur Flow Cytometer (Becton‐Dickinson, San Jose, CA). Cell cycle and apoptosis were calculated by gating analysis. At least 10,000 cells were analysed per sample.

### 2.11 Wound healing and invasion assay

5 × 10^5^ cells were seeded in 6‐well plates and incubated until they reached 90% confluence. The confluence plates were scratched using a sterile pipette tip, and photographed under a microscope at 0, 24 h and 48 h. Cell migration was measured by monitoring the width of the scratch over time.

Invasion assays were performed in a 24‐well transwell chamber. Briefly, cells suspended in 200 μl of serum‐free media were placed in the upper Matrigel chamber (coated with 15 μg of Matrigel). Another 500 μl of medium supplemented with 10% FBS was placed in the lower chamber. Cells that migrated through the membrane after 24 h were stained, photographed, and counted with Giemsa (Sigma, St. Louis, MO).

### 2.12 Immunohistochemistry analysis

Liver cancer chip HLivH060CD03 was purchased from Shanghai Outdo Biotech Co., Ltd. Immunostaining was performed by using the Vectastain ABC Kit. According to the manufacturer's instructions, the chip was incubated with SSR2 primary antibody (1:500; Proteintech, Chicago, US) at 4°C overnight, and subsequently with HRP‐conjugated polymers for 20 min. After DAB visualization and haematoxylin counterstaining, 5 fields per slide were scored. Staining in brown was considered positive.

### 2.13 In vivo subcutaneous tumour growth xenograft

BALB/c‐nude mice were obtained from Basic Medicine Laboratory of 900 Hospital. All animal experimental protocols were approved by the Animal Investigation Committee of 900 Hospital of the Joint Logistics Support Force. HepG2‐shSSR2‐2 or HepG2‐shCtrl cells (6 × 10^6^) were suspended in PBS with 50% Matrigel and injected into the left flank of the nude mice. The tumour volume and mice body weight were measured after 29 days. Tumour volume was calculated as (length × width × height × π)/6. At the end of experiment, all the mice were sacrificed, and the weight of solid tumours were recorded.

### 2.14 Statistical analysis

Results were expressed as the mean ± standard deviation. SPSS Statistics (version 21.0) and the GraphPad Prism (version 7.0) software were employed for statistical analysis and visualization. Independent sample T‐test and one‐way ANOVA were used for statistical analyses between groups of continuous variables that followed the normal distribution. Kaplan–Meier survival analysis was also performed for overall prognostic analyses. *p*‐values <0.05 were considered statistically significant.

## 3 RESULTS

### 3.1 High expression of SSR2 in HCC

**FIGURE 1:**
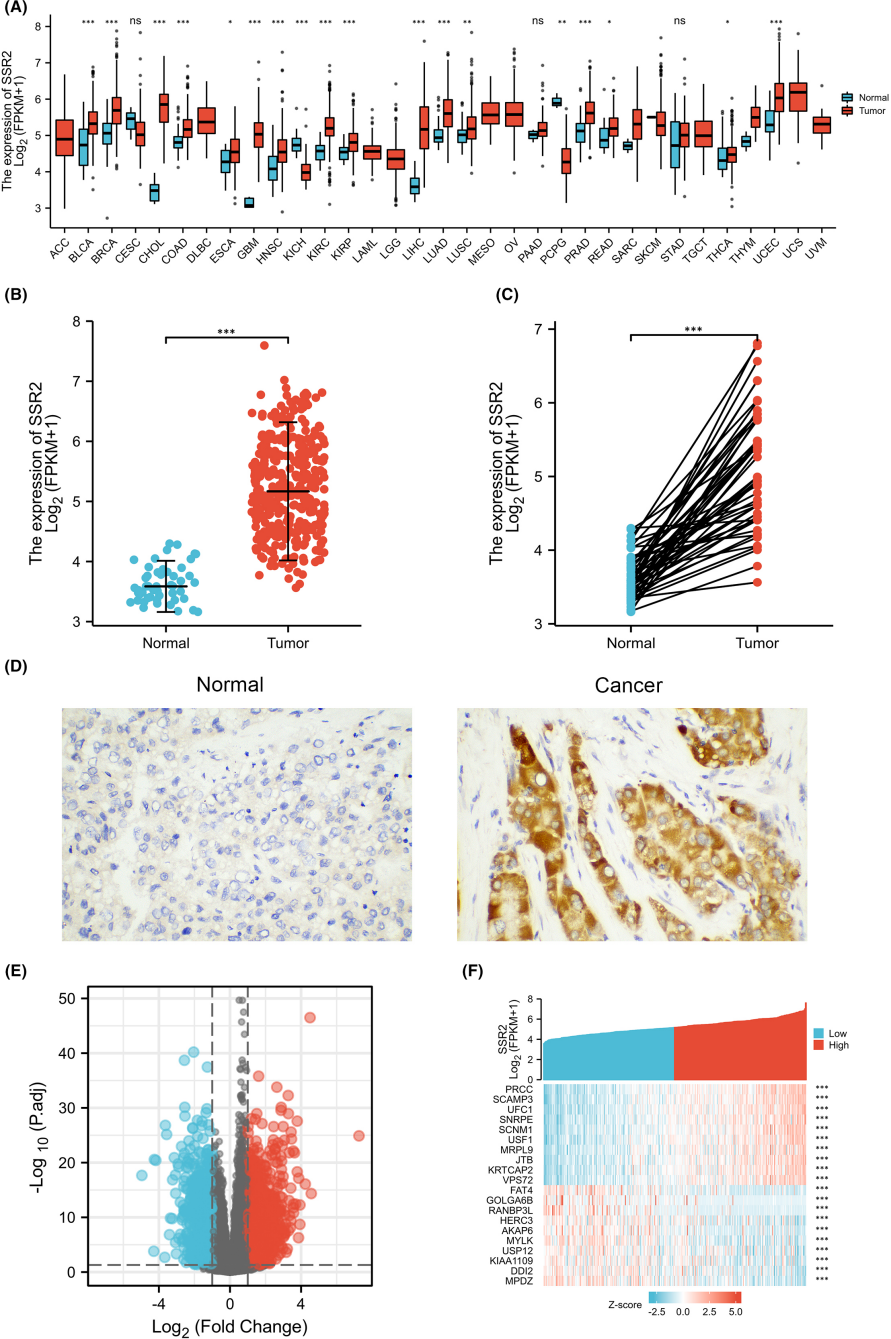
Differential expression levels of SSR2 in different malignancies and SSR2‐related differentially expressed genes (DEGs). (A) dysregulated SSR2 expressions of different cancers compared with adjacent normal tissues in the TCGA database. (B and C) Differential expression levels of SSR2 in HCC. (D) Immunohistochemistry analysis of SSR2 expression in HCC and adjacent normal samples. ×200 magnifications: the samples of HCC show strong expression of SSR2. (E and F) Volcano plots of the DEGs between SSR2‐high and SSR2‐low cohorts and heat map showing the top 20 DEGs

As shown in Figure 1A, SSR2 was significantly dysregulated in bladder urothelial carcinoma (BLCA), breast infiltrating carcinoma (BRCA), cholangiocarcinoma (CHOL), colon adenocarcinoma (COAD), oesophageal carcinoma (ESCA), pleomorphic glioma (GBM), head and neck squamous cell carcinoma (HNSC), renal chromophobe cell carcinoma (KICH), renal clear cell carcinoma (KIRC), renal papillary cell carcinoma (KIRP), liver hepatocellular carcinoma (LIHC), lung adenocarcinoma (LUAD), lung squamous cell carcinoma (LUSC), pheochromocytoma and paraganglioma (PCPG), prostate cancer (PRAD), Lung squamous cell carcinoma (LUSC), thyroid cancer (THCA), rectum adenocarcinoma (READ) and endometrial cancer (UCEC) (*p < 0.05; **p < 0.01; ***p < 0.001). In 50 paracancerous and 374 HCC samples in TCGA‐LIHC dataset, the expression of SSR2 was significantly higher in HCC (***p < 0.001) (Figure 1B). Meanwhile, there was significant difference between the expression of SSR2 in 50 HCC and matched paracancerous samples (***p < 0.001) (Figure 1C). Differential expression of SSR2 was found in 17 paired HCC and adjacent tissues and normal adjacent tissues by immunohistochemistry analysis (Figure 1D).

### 3.2 Identification of DEGs associated with SSR2 in HCC

To identify the DEGs associated with SSR2 in HCC, 187 HCC SSR2‐high samples were compared with 188 SSR2‐low controls, and a total of 952 DEGs, covering 649 upregulated genes and 303 downregulated genes, were determined to be statistically significant (Figure 1E, Table S1). The heatmap of the relative expression values for the top 20 DEGs between SSR2‐high and SSR2‐low cohorts were also showed in Figure 1F.

### 3.3 Association between SSR2 expression and clinicopathological parameters

**FIGURE 2:**
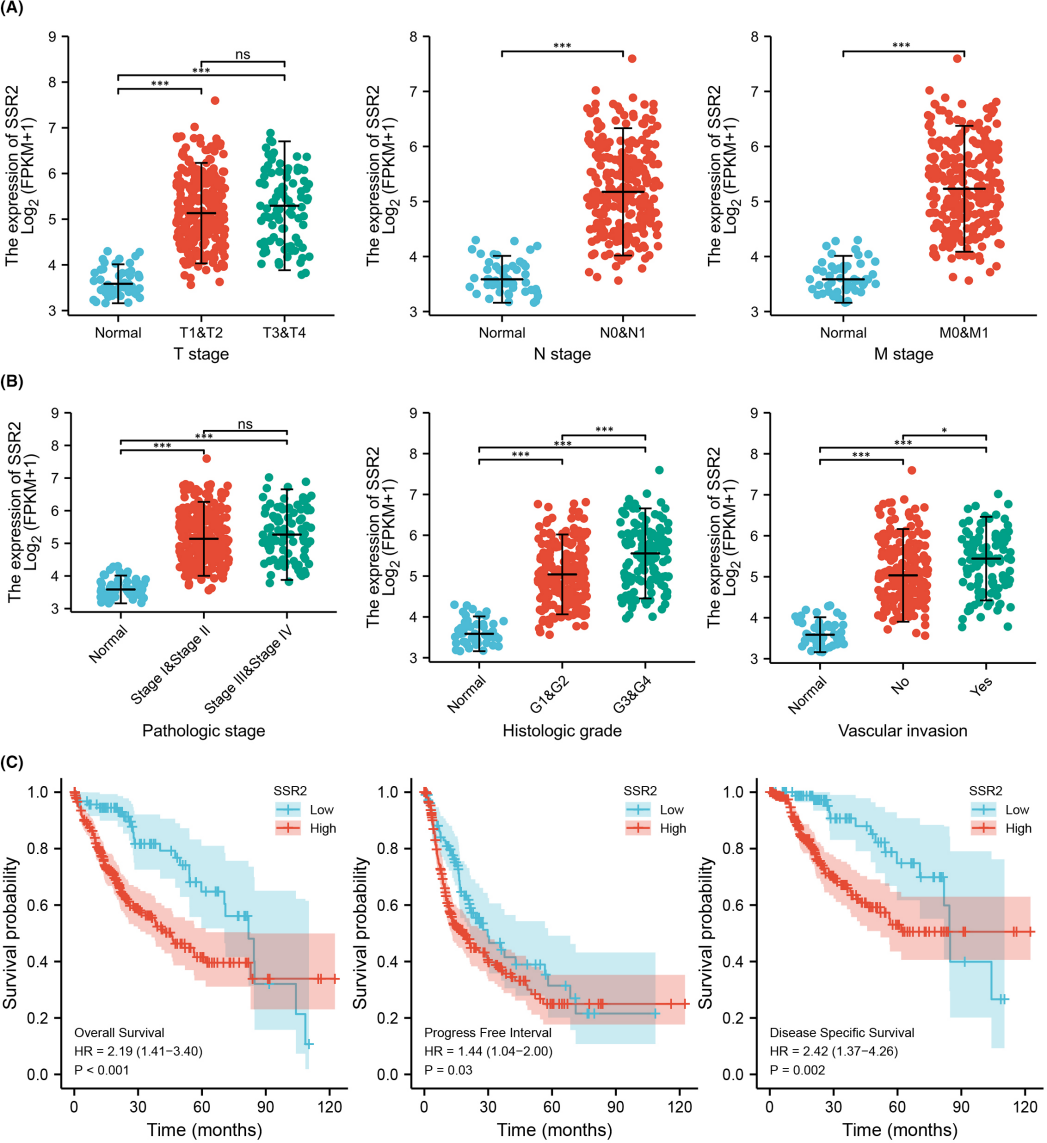
Association between SSR2 expression and clinicopathological characteristics. (A) T stage, N stage, and M stage, (B) pathologic stage, histological grade, and vascular invasion. (C) Survival curves of OS, DSS, and PFI between SSR2‐high and SSR2‐low patients with GC in TCGA cohort. ***p < 0.001, *p < 0.05

To clarify the role and significance of SSR2 in HCC progression, a total of 368 HCC samples with SSR2 expression data and all the patients’ characteristics were analysed from TCGA‐LIHC. As shown in Figure 2A and B and Table 1, the overexpression of SSR2 was significantly correlated with T stage (t1 & 2 vs. normal, t3 & 4 vs. normal; ***p < 0.001), N stage (n0 & 1 vs. normal; ***p < 0.001), M stage (m0 & 1 vs. normal; ***p < 0.001), pathological stage (stage1 & 2 vs. normal, stage 3 & 4 vs. normal; ***p < 0.001), histological grade (grade 1 & 2 vs. normal, grade 3 & 4 vs. normal; ***p < 0.001), vascular invasion (No vs. normal, yes vs. normal, ***p < 0.001; yes vs. no, *p < 0.05). We also univariately analysed the logistic regression illuminated SSR2 expression (Table 2). Increased SSR2 expression in HCC is positively associated with T stage (OR = 1.889 for T2 & T3 & T4 vs. T1, *p* = 0.003), histological grade (OR = 2.669 for G3 & G4 vs. G1 & G2, *p* < 0.001) and vascular invasion (OR = 2.314 for Yes vs. No, *p* < 0.001). The 120‐month OS rates were significantly lower in patients with high SSR2 expression than those with low SSR2 expression (*p* < 0.001). Similarly, the 10‐year PFI rates (*p* = 0.03) and DSS (*p* = 0.002) in the SSR2‐low group were significantly higher than those in the SSR2‐high group. Subgroup survival analyses of OS, DSS, and PFI were shown in Figure S1. All the results above suggested that HCCs with high SSR2 expression were more likely to progress to a more advanced stage than those with low SSR2 expression.

**TABLE 1:** Association between SSR2 expression and clinicopathological variables

Characteristic	Low expression of SSR2	High expression of SSR2	*p*
*n*	185	186	
Gender, *n* (%)			0.016
Female	49 (13.2%)	72 (19.4%)	
Male	136 (36.7%)	114 (30.7%)	
Age, *n* (%)			0.022
≤60	77 (20.8%)	100 (27%)	
>60	108 (29.2%)	85 (23%)	
BMI, n (%)			0.147
≤25	79 (23.6%)	98 (29.3%)	
>25	84 (25.1%)	74 (22.1%)	
Child‐Pugh score, *n* (%)			0.366
A	109 (45.6%)	108 (45.2%)	
B	13 (5.4%)	8 (3.3%)	
C	1 (0.4%)	0 (0%)	
AFP (ng/ml), *n* (%)			<0.001
≤400	126 (45.3%)	87 (31.3%)	
>400	11 (4%)	54 (19.4%)	
Albumin (g/dl), *n* (%)			0.636
<3.5	38 (12.8%)	31 (10.4%)	
≥3.5	116 (39.1%)	112 (37.7%)	
Prothrombin time, *n* (%)			0.053
≤4	97 (33%)	109 (37.1%)	
>4	53 (18%)	35 (11.9%)	
T stage, *n* (%)			0.019
T1	105 (28.5%)	76 (20.7%)	
T2	38 (10.3%)	56 (15.2%)	
T3	34 (9.2%)	46 (12.5%)	
T4	7 (1.9%)	6 (1.6%)	
N stage, *n* (%)			1.000
N0	124 (48.4%)	128 (50%)	
N1	2 (0.8%)	2 (0.8%)	
M stage, *n* (%)			0.626
M0	124 (45.9%)	142 (52.6%)	
M1	1 (0.4%)	3 (1.1%)	
Pathological stage, *n* (%)			0.035
Stage I	98 (28.2%)	73 (21%)	
Stage II	34 (9.8%)	52 (15%)	
Stage III	39 (11.2%)	46 (13.3%)	
Stage IV	2 (0.6%)	3 (0.9%)	
Histological grade, *n* (%)			<0.001
G1	36 (9.8%)	19 (5.2%)	
G2	101 (27.6%)	76 (20.8%)	
G3	44 (12%)	78 (21.3%)	
G4	3 (0.8%)	9 (2.5%)	
Fibrosis ishak score, *n* (%)			0.557
0	43 (20.3%)	31 (14.6%)	
1/2	18 (8.5%)	13 (6.1%)	
3/4	12 (5.7%)	16 (7.5%)	
5/6	43 (20.3%)	36 (17%)	
Vascular invasion, *n* (%)			<0.001
No	120 (38.1%)	86 (27.3%)	
Yes	41 (13%)	68 (21.6%)	

**TABLE 2:** SSR2 expression association with clinical pathological characteristics (logistic regression)

Characteristics	Total (*N*)	Odds ratio (OR)	*p*‐value
T stage (T2 & T3 & T4 vs. T1)	368	1.889 (1.251–2.865)	0.003
N stage (N1 vs. N0)	256	0.969 (0.115–8.176)	0.975
M stage (M1 vs. M0)	270	2.620 (0.331–53.331)	0.407
Pathological stage (Stage III & Stage IV vs. Stage I & Stage II)	347	1.262 (0.780–2.049)	0.344
Tumour status (With tumour vs. Tumour free)	352	1.456 (0.954–2.230)	0.083
Gender (Male vs. Female)	371	0.570 (0.366–0.884)	0.012
Age (>60 vs. ≤60)	370	0.606 (0.401–0.913)	0.017
Height (≥170 vs. <170)	339	0.491 (0.316–0.761)	0.002
BMI (>25 vs. ≤25)	335	0.710 (0.461–1.091)	0.119
Residual tumour (R1 & R2 vs. R0)	342	1.250 (0.481–3.351)	0.647
Histological grade (G3 & G4 vs. G1 & G2)	366	2.669 (1.725–4.171)	<0.001
AFP (ng/ml) (>400 vs. ≤400)	278	7.110 (3.641–15.047)	<0.001
Child‐Pugh score (B & C vs. A)	239	0.577 (0.222–1.403)	0.235
Fibrosis ishak score (1/2 & 3/4 & 5/6 vs. 0)	212	1.235 (0.700–2.195)	0.468
Vascular invasion (Yes vs. No)	315	2.314 (1.443–3.747)	<0.001

### 3.4 GSEA analysis identifies SSR2‐related signalling pathways

**FIGURE 3:**
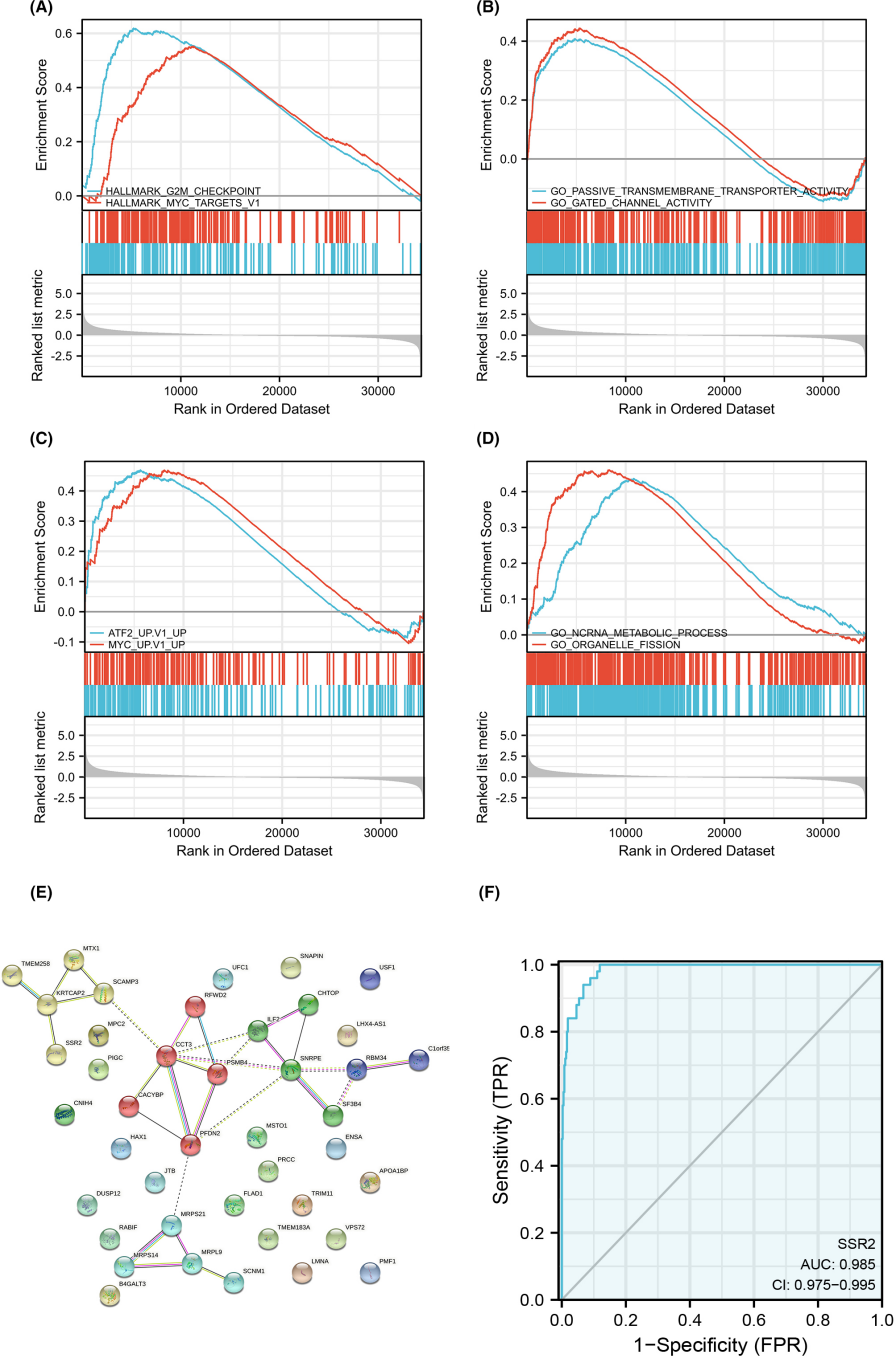
GSEA analysis of SSR2‐related genes in HCC. (A) G2/M checkpoint, (B) passive transmembrane transporter activity, (C) ATF2_S_UP. V1_UP and (D) ncRNA metabolic process. NES, normalized enrichment score; *p*‐adj, adjusted *p*‐value; FDR, false discovery rate. (E) Construction of PPI network associated with SSR2 in HCC. (F) A ROC curve to test the value of SSR2 to identify HCC tissues was created

To identify SSR2‐related signalling pathways in HCC, SSR2‐high and SSR2‐low expression data sets were enriched with the MSigDB Collection (h.all.v7.2.symbols.gmt, c6.all.v7.2.symbols.gmt, etc.) to reveal significant differences (false discovery rate [FDR] < 0.25 and *p*‐adjust <0.05). GSEA analysis showed 4 significant KEGG pathways associated with risk score, including G2/M checkpoint, passive transmembrane transporter activity, ATF2_S_UP. V1_UP and ncRNA metabolic process (Figure 3A–D, Table S2).

### 3.5 PPI network and ROC analysis associated with SSR2

To investigate the interactions between the top DEGs in HCC, the Search Tool for the Retrieval of Interacting Genes/Proteins database (STRING v10.5) was employed to construct a PPI network associated with SSR2 (Figure 3E). All the interactions between them were derived from high‐throughput laboratory experiments and previous knowledge in curated databases at high level of confidence (sources: experiments, databases; score ≥ 0.90). Additionally, top hub genes included TMEM258, KRTCAP2, MTX1, SCAMP3, CCT3, RFWD2, PSMB4, CACYBP, and PFDN2, etc. ROC was also used to analyse the distinguishing efficacy of SSR2 between HCC and normal liver tissues. The area under the curve (AUC) of SSR2 is 0.985 (CI: 0.975–0.995), suggestive of a potential identifier for HCC. (Figure 3F).

### 3.6 SSR2 promotes liver cancer cell proliferation in vitro

**FIGURE 4:**
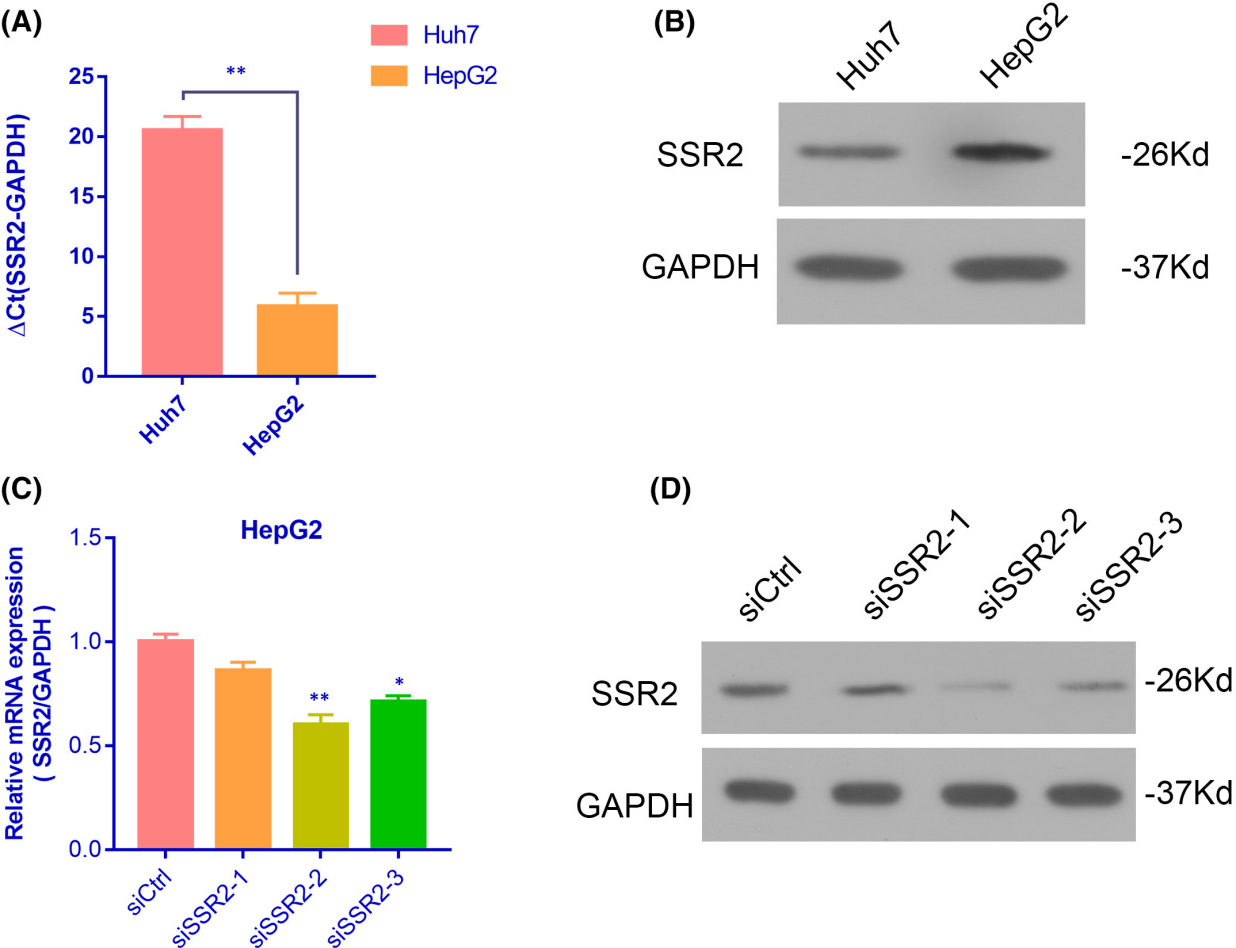
Small interference mediated SSR2 downregulation is effective in HCCs. (A and B) The mRNA expression level of SSR2 was detected with qRT‐PCR and Western blotting in two HCC cells. Histogram is the average value (mean ± SD) of three independent experiments. Small interference mediated SSR2 downregulation was also determined by by qRT‐PCR and Western blotting in HEPG2 cells (C and D). All were done at least three independent experiments. **p < 0.001

**FIGURE 5:**
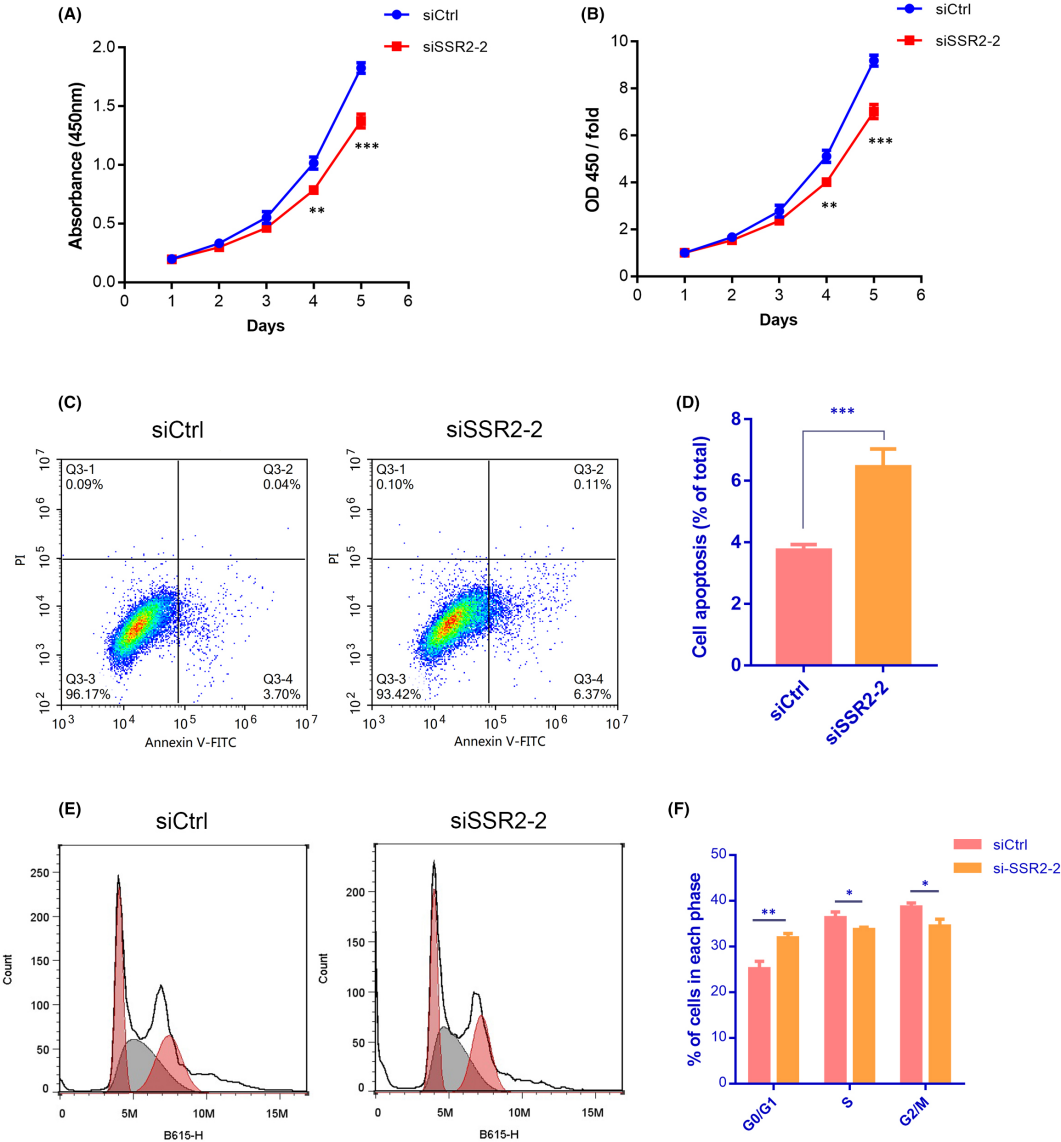
Downregulation of SSR2 inhibits the proliferation and promotes apoptosis and cell cycle arrest of HepG2 cells. (A and B) Statistical result of cell count/fold and growth curve of siCtrl and siSSR2‐2 groups. Data are presented as the mean ± SD from three independent experiments. **p < 0.01, ***p < 0.001. (C and D) Apoptosis ratios of siSSR2‐2 group were increased compared with those in siCtrl group. Histogram is the average cell apoptosis rate (mean ± SD) of three independent experiments. ***p < 0.001. (E and F) S and G2/M phases of siSSR2‐2 groups were decreased compared with those in siCtrl group. Histogram is the average ratio (mean ± SD) of three independent experiments. *p < 0.05, **p < 0.01 by Student's *t*‐test

ARCHS^4^ database revealed that SSR2 was abundantly expressed in liver cells including HepG2 (Figure S2). Further research found that both SSR2 mRNA and protein were highly expressed in HepG2 cells than in Huh‐7 cells compared with internal reference gene GAPDH. (Figure 4A and B). After transfected with SSR2‐siRNA, the mRNA and protein expression of SSR2 was downregulated significantly in HepG2 cells (Figure 4C and D). Using MTT assay, we found that cell proliferation rates were significantly reduced at different time points from Day 1 to Day 5, compared with siCtrl group (Figure 5A and B, **p < 0.01, ***p < 0.001). Flow cytometry showed that number of apoptotic and necrotic cells was elevated significantly after siSSR2‐2 transfection (Figure 5C and D, ***p < 0.001). Cell cycle experiment showed that SSR2 depletion significantly decreased the percentage of HepG2 cells in S‐phase and G2/M‐phase (Figure 5E and F right panel, *p < 0.05, **p < 0.01). It suggested that SSR2 played an essential role in the proliferation of HCC cells.

### 3.7 SSR2 depletion inhibits cell migration and invasion in vitro

**FIGURE 6:**
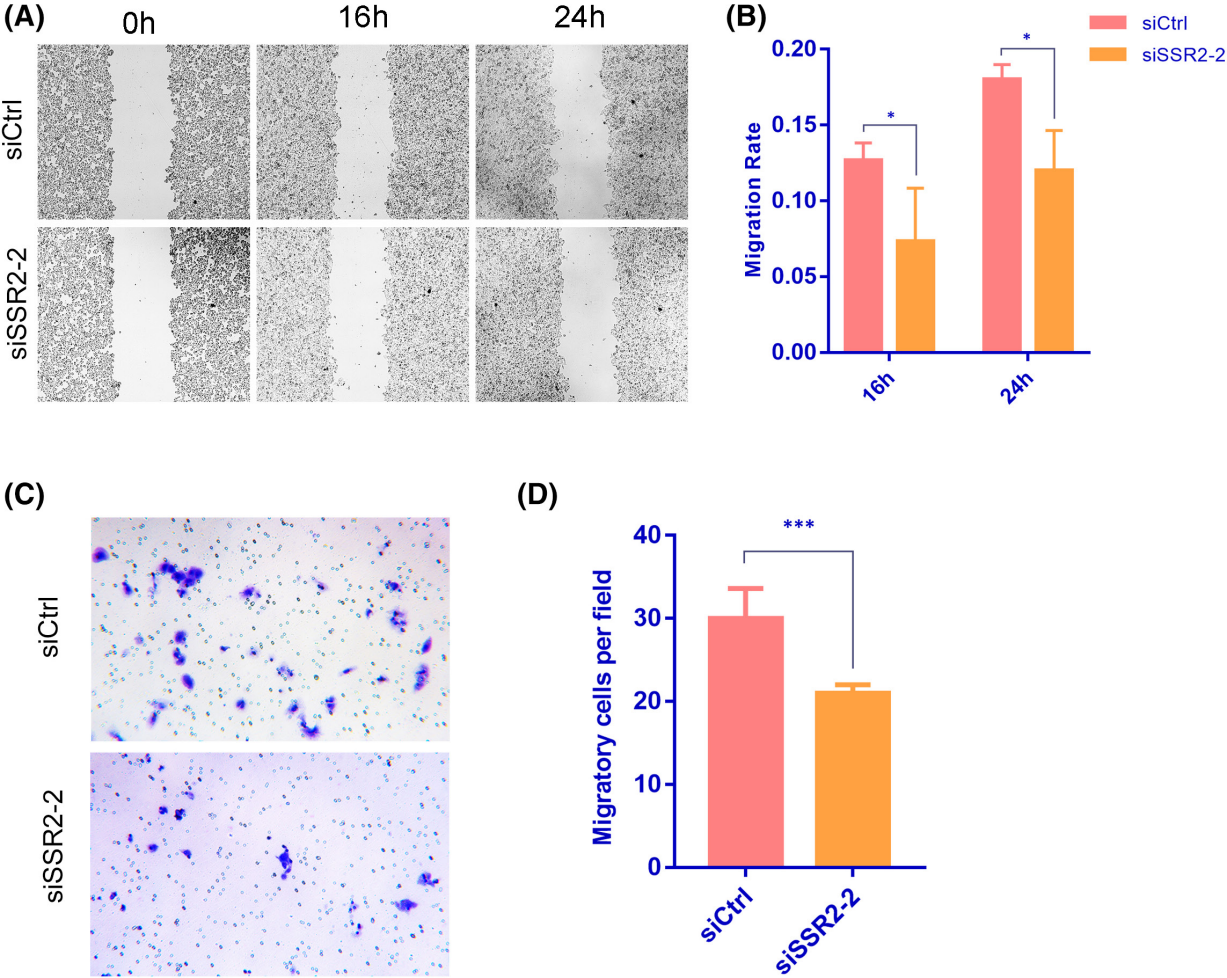
Effect of siSSR2‐2 transfection on the migration and invasion of HepG2 cells. (A and B) Cell migration after siSSR2‐2 transfection analysed by wound healing assay, as compared with siCtrl group. (C and D) Invaded HepG2 cells analysed by Transwell assay, as compared with siCtrl group. The results are presented as means ± SD, and data from three independent experiments are shown. *p < 0.05, *** *p* < 0.001 compared by *t*‐test with siCtrl‐transfected HepG2 cells

Downregulation of SSR2 notably inhibited cell migration in the wound assay (Figure 6A and B). In addition, the number of migrating HepG2 cells in the Transwell assay was significantly decreased in the siSSR2‐2 group compared with the siCtrl group (Figure 6C and D, ***p < 0.001). These results suggested that SSR2 played a role in promoting HCC cell migration.

### 3.8 Downregulation of SSR2 represses the tumour growth of HepG2 cells in vivo

**FIGURE 7:**
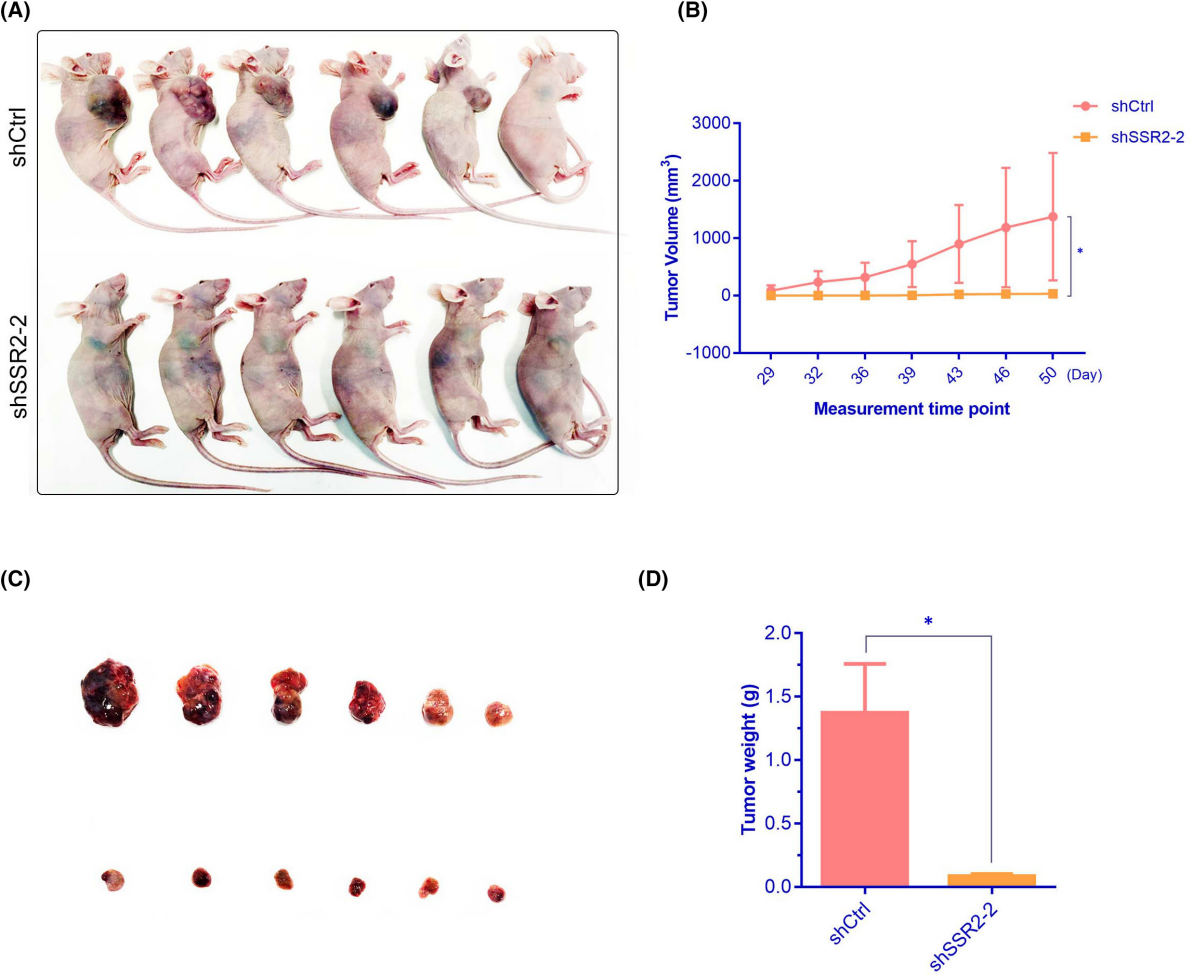
Effect of SSR2 on tumour growth in vivo. (A) Images represent tumour growth in the nude mice 50 days after they were injected with 5 × 10^6^ shCtrl or shSSR2‐2 stable HepG2 cells (*n* = 6 mice/group). (B) Tumour volume was measured every 3 or 4 days and calculated. Data are mean ± SE (*n* = 6). *p < 0.05. (C) Represented images of tumours from each group. (D) Tumour weight. Data are mean ± SE

A rodent model was used to detect the growth of HepG2 cell tumors with or without expression of SSR2. Tumours in nude mice were markedly smaller in the shSSR2‐2 group compared with those in the siCtrl group (Figure 7A). RT‐PCR result showed that the expressions of SSR2 was also significantly downregulated in shSSR2‐2 group tumour samples compared with shCtrl group (Figure S3). Tumour volumes and weights were also significantly lower in the shSSR2‐2 group (Figure 7B–D, *p < 0.05). These results suggested that silencing SSR2 inhibited HCC growth in vivo.

## 4 DISCUSSION

To our knowledge, early stage HCC may be treated by a variety of methods, including surgical resection, radiofrequency ablation and chemoembolization. However, among patients with HCC, only 20% receive surgical treatment, and the 5‐year survival of patients after surgical resection was not ideal, and >70% of patients relapsed within 5 years. Meanwhile, more than 80% of patients were found to be at an advanced stage, the treatment options are limited and the prognosis is poor. Exploiting novel therapeutic targets is imperative to improve the survival of HCC patients.

In this study, using various bioinformatics methods, we found that an increased expressions of SSR2 in HCC were associated with clinicopathological characteristics, shorter survival time and poorer prognosis. To further investigate the possible functions of SSR2 in HCC progression, we performed GESA and PPI network analyses. The results revealed that G2/M checkpoint, passive transmembrane transporter activity, ATF2_S_UP. V1_UP and ncRNA metabolic process were differentially enriched in the SSR2‐high phenotype. PPI network analysis also revealed the top hub genes TMEM258, KRTCAP2, and MTX1. The AUC of SSR2 for ROC was 0.985. These data suggested that SSR2 might serve as a potential prognostic marker and a therapeutic target in HCC. In vitro, consistent with previous study,^11^ we also found that SSR2 silencing inhibited proliferation and promoted cellular apoptosis and cell cycle arrest in HepG2 cells. Moreover, SSR2 silencing was also demonstrated to reduce HCC cell migration and invasion. The in vivo study further demonstrated that SSR2 promoted the growth of HCC cells in xenograft mouse models.

Although the above results improved our understanding of the relationship between SSR2 and HCC, there were some limitations. Firstly, the retrospective studies of TCGA‐LIHC lacked of some patient information, which may influence the results we have obtained. Secondly, the possible signalling pathways associated with SSR2 need to be further elucidated.

In this study, we reported that SSR2 was highly expressed in HCC tissues and its high expression was significantly associated with the progression, poor survival, which might promote tumorigenesis through G2/M checkpoint. In vitro study confirmed that SSR2 knockdown via siRNA transfection inhibited cell proliferation, migration, invasion ability and promoted apoptosis, cell cycle arrest. SSR2 depletion also repressed the tumour growth of HCC cells in vivo. Therefore, SSR2 may become a new biomarker of HCC and has the potential to predict treatment outcomes. The mechanism of SSR2 promoting the progression and metastasis of HCC will be verified in further studies.

## 5 CONCLUSION

Taken together, our study establishes the role for SSR2 dysregulation in HCC. We fully elucidated its expression profiles, survival analysis and the potential signalling associated with SSR2. Additionally, we report for the first time that the oncogenic activity of SSR2 is attributable to the promotion of HCC cell proliferation, invasion, migration and the inhibition of cell apoptosis. Nonetheless, in this study, we only focus on the cellular function and possible signalling pathways of SSR2 in HCC. Thus, future mechanism of SSR2 is required to uncover. Only by completely elucidating the molecular mechanisms of SSR2 in HCC can we open avenues for utilizing SSR2 to identify novel diagnostic or therapeutic target.

## CONFLICT OF INTEREST

No potential conflict of interest was reported by the authors.

## AUTHOR CONTRIBUTIONS

**Fengsui Chen:** Formal analysis (lead); Investigation (lead); Writing – original draft (lead). **Xingchi Chen:** Data curation (supporting); Investigation (supporting). **Jielong Wang:** Investigation (supporting). **Shian Zhang:** Conceptualization (supporting); Methodology (supporting). **Mengxue Chen:** Conceptualization (lead); Investigation (supporting). **Xia Zhang:** Conceptualization (lead); Methodology (lead). **Zhixian Wu:** Conceptualization (supporting); Funding acquisition (lead); Investigation (lead).

## Supporting information


Fig S1

Fig S2

Fig S3

Table S1

Table S2
