# Supplementary figures and images for: Overexpression of SSR2 promotes proliferation of liver cancer cells and predicts prognosis of patients with hepatocellular carcinoma
Source: J Cell Mol Med. 2022 Apr 28;26(11):3169–82. doi: 10.1111/jcmm.17314 (PMC9170819; doi:10.1111/jcmm.17314)

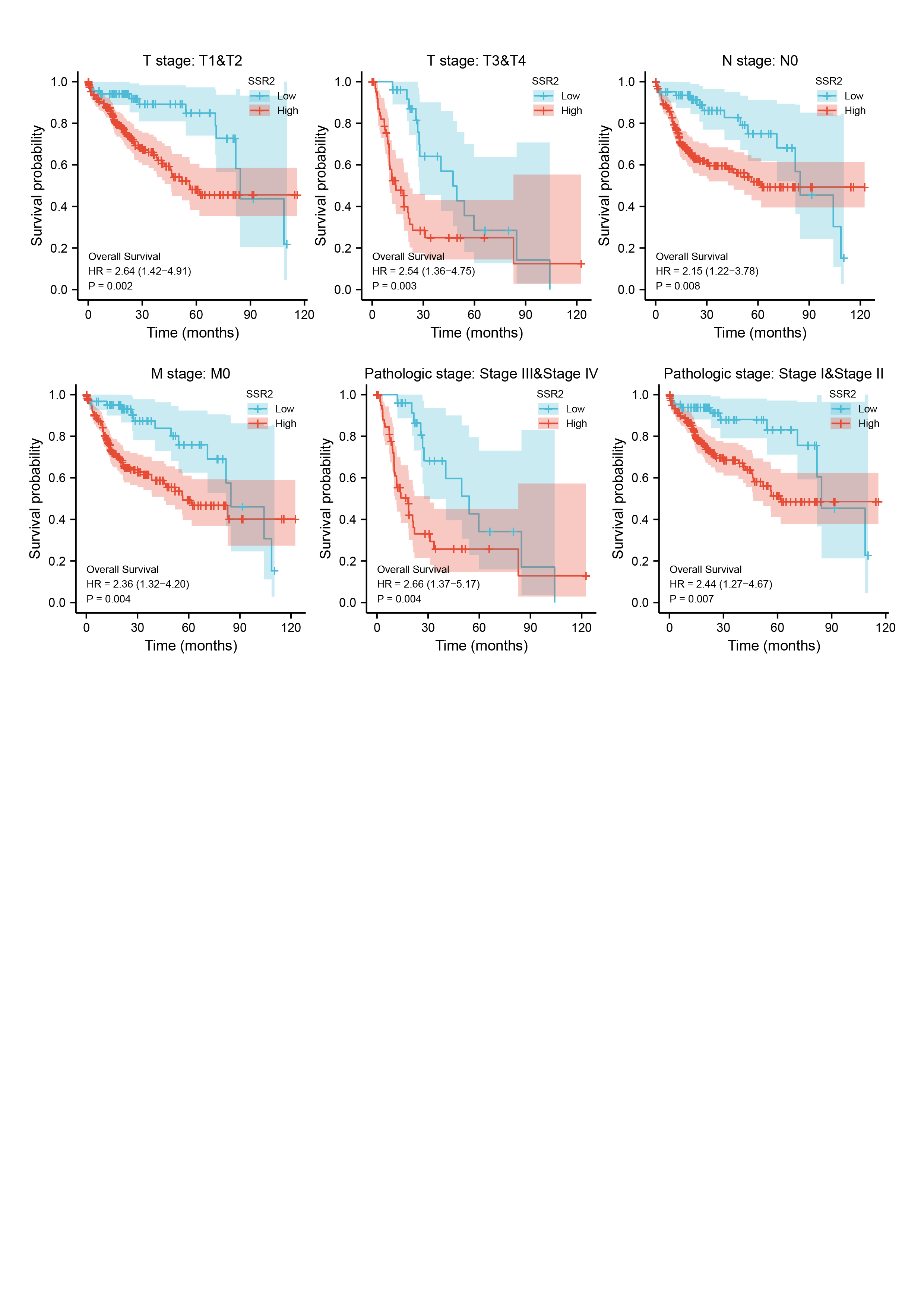

Supplement: Supplementary file 1 — Fig S1 [file JCMM-26-3169-s002.jpg]

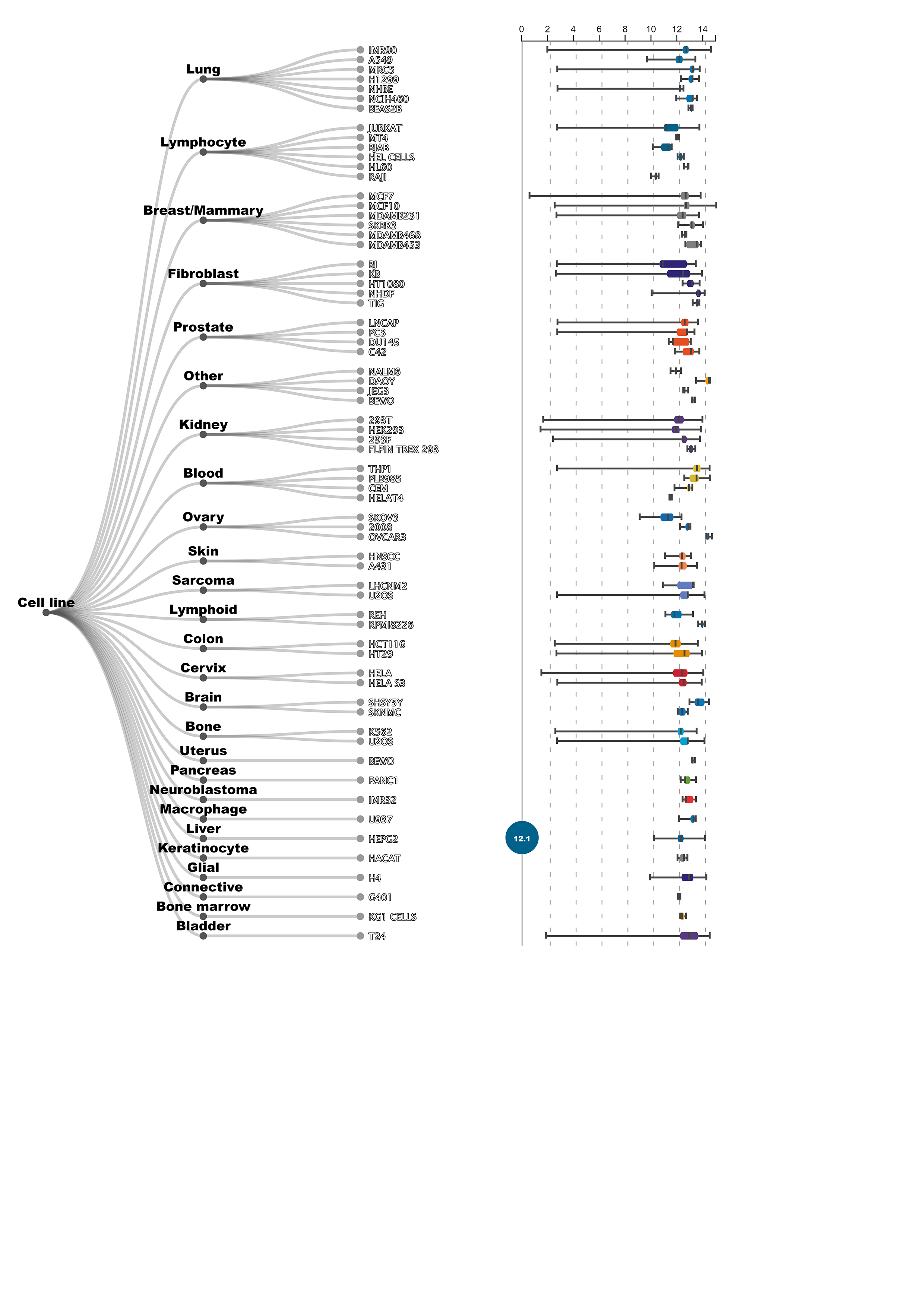

Supplement: Supplementary file 2 — Fig S2 [file JCMM-26-3169-s005.jpg]

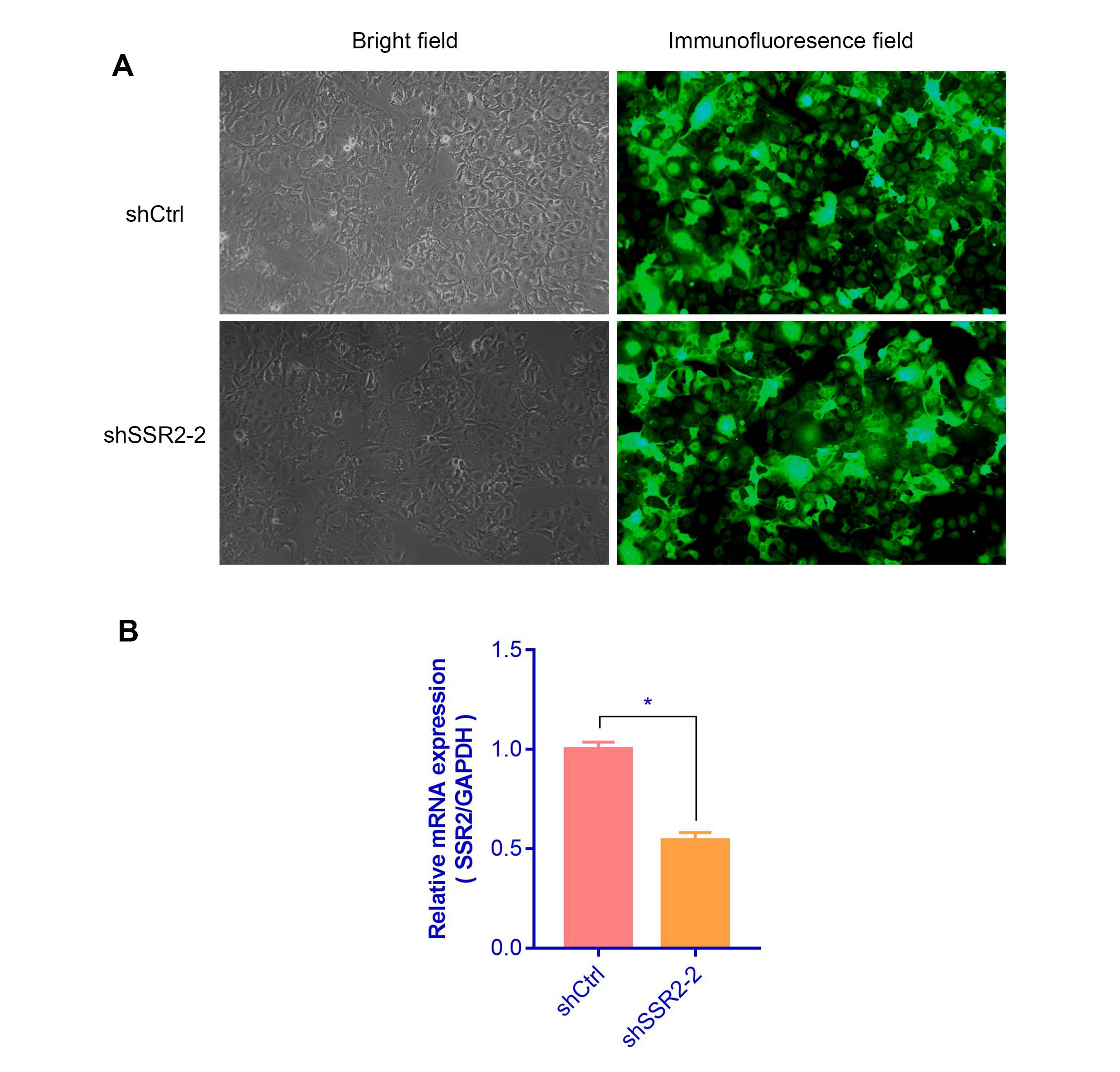

Supplement: Supplementary file 3 — Fig S3 [file JCMM-26-3169-s004.jpg]
